# Reduction of Activin Receptor-Like Kinase 4 Expression Ameliorates Myocardial Ischemia/Reperfusion Injury through Inhibiting TGF*β* Signaling Pathway

**DOI:** 10.1155/2022/5242323

**Published:** 2022-03-31

**Authors:** Mantian Chen, Yinggang Sun, Qian Wang, Yigang Li

**Affiliations:** Department of Cardiology, Xinhua Hospital, School of Medicine, Shanghai Jiao Tong University, Shanghai 200092, China

## Abstract

The activation of activin receptor-like kinase 4 (ALK4) signaling plays a pivotal role in the pressure-overloaded heart, and haplodeficiency of ALK4 can alleviate cardiac fibrosis secondary to myocardial infarction and preserve cardiac function through partially inactivating the Smad3/4 pathway. However, whether transforming growth factor (TGF) *β* signaling is involved in the beneficial effects of ALK4 knockdown on the ischemic heart is still unclear. This study was undertaken to investigate the change in the TGF*β* signaling after ALK4 knockdown *in vivo* and in vitro. Forty C57BL/6J mice were randomized into ALK4^+/-^ ischemia/reperfusion (I/R) group (ALK4^+/-^+I/R, *n* = 10), ALK4^+/-^ sham group (ALK4^+/-^+sham, *n* = 10), wild-type sham group (WT+sham, *n* = 10), and WT I/R group (WT+I/R, *n* = 10). Heart histology and the levels of cytokines related to antioxidant and inflammation, as well as protein and mRNA expressions of molecules associated with TGF*β* pathway, were examined in different groups. Our results showed that the reduction of ALK4 expression ameliorated myocardial I/R injury through inhibiting TGF*β* signaling pathway. Our findings indicate that ALK4 may become a novel target for the therapy of myocardial I/R injury.

## 1. Introduction

Ischemia/reperfusion (I/R) injury is still a challenge in clinical practice [1], and cardiomyocyte apoptosis is one of the main pathophysiological processes in the myocardial I/R injury [2–4]. I/R injury may aggravate the structural remodeling and dysfunction of ischemic myocardium after acute myocardial infarction [5]. Thus, it is imperative to develop effective therapeutic goals for protecting the heart against the I/R injury.

Studies have confirmed that transforming growth factor *β* (TGF*β*) is involved in diverse cellular processes: proliferation, differentiation, and matrix formation. TGF*β* is important in the regulation of tissue homeostasis [6, 7]. TGF*β* can bind to distinct receptors of the activin receptor-like kinase (ALK) family, which then induces the phosphorylation of receptor-regulated Smad (R-Smad) proteins (such as Smad1/2/3/5/8) at the intracellular carboxy terminal (C terminal). The phosphorylated R-Smads subsequently form complexes with the common mediator Smad (C-Smad), such as Smad4. Then, these complexes, together with Smad4, translocate to the nucleus and bind to DNA to regulate the transcription of some genes via recruiting transcription factors [8, 9], such as Cyclin D1 [10], c-Myc [11], and c-jun [12]. Increasing evidence demonstrates that TGF*β*1 is dysregulated in the heart, brain, kidney, intestine, and other organs experiencing I/R injury, indicating that TGF*β*1 plays a crucial role in the progression of I/R injury [13–16].

ALK4 is a type I receptor of TGF*β*, can be specifically activated by activin, and is able to mediate some biological processes such as cell differentiation, proliferation, and migration [17]. Studies have indicated that ALK4 can inhibit cell growth and induce fibrosis in the liver [18], skin, and kidney [19]. Our previous study showed that haplodeficiency of ALK4 alleviated cardiac fibrosis secondary to myocardial infarction and preserved cardiac function [20], and partial inhibition of ALK4 was able to attenuate cardiac fibrosis result from the pressure overload and improve cardiac function [21] through suppressing Smad2/3 activity. However, whether the beneficial effects of ALK4 knockdown on the ischemic heart are also mediated by the TGF*β* signaling is still poorly understood. The present study was aimed at investigating if TGF*β* signaling was involved in the functions of ALK4 knockdown on the myocardial I/R injury. Our findings will provide evidence for new approaches for the treatment of myocardial I/R injury.

## 2. Materials and Methods

### 2.1. Experimental Ethics Policy

All the experimental procedures were performed in accordance with the guidelines from the Committee for the Care and Use of Laboratory Animals. This study was approved by the local committee on animal research.

### 2.2. Animals

Animal research was approved by the Animal Care and Use Committee of the Shanghai Xinhua Hospital, School of Medicine, Shanghai Jiao Tong University. C57BL/6J mice (18–22 g; 6-8 weeks) were purchased from the Experimental Animal Center of the Peking University (Beijing, China). As mentioned earlier, the USE CRISPR-Cas9 technique was used to produce ALK4 monoploid defect mice (ALK4). All mice were housed with the temperature at 18–22°C, the relative humidity of 50–70%, 12-hour light/dark cycle before the experiment. These were assigned to four experimental groups: wild type (WT)+sham group, WT+I/R group, ALK4^+/-^+sham group, and ALK4^+/-^+I/R group (*n* = 10 per group). The I/R model was established in the morning (08 : 00-12 : 00).

### 2.3. Establishment of Myocardial I/R Injury Model

The myocardial I/R injury model was established according to the following procedures as previously reported [21]. Briefly, mice were injected with heparin at 1,000 IU/kg (i.p.) and anesthetized with sodium pentobarbital at 60 mg/kg (i.p.), 5 min later. Sodium pentobarbital (50 *μ*l; 20 mg/kg, i.p.) was administered if necessary. Then, the mouse was intubated with a 19G stump needle and then ventilated by using a mouse ventilator (Mini-Vent) with the respiratory rate at 210 breaths/min. Following left thoracotomy at the fourth intercostal space, the left anterior descending coronary artery (LCA) was exposed and ligated using a 6.0 prolene suture under a microscope. The regional ischemia was confirmed by the presence of discoloration of the occluded distal myocardium on visual inspection under a microscope. The ligation was released to induce reperfusion after 45 min ischemia, and the reperfusion was also confirmed by visual inspection [21].

### 2.4. Heart Histology

The hearts were collected, fixed in formalin overnight, embedded in paraffin, and sectioned. After hematoxylin and eosin (HE) staining, the size of myocytes in the left ventricle (LV) was measured. For the detection of cardiac infarct size, the LCA was occluded again at the same site after perfusion, and 2% Evans blue dye (0.3 ml) was injected into the abdominal vein, followed by identification of the area at risk. The nonischaemic myocardium was blue. The heart was collected, frozen at -20°C for 20 min, and sliced transversely (1 mm in thickness). The slices were incubated in 2% 2,3,5-triphenyltetrazolium chloride (TTC) (Sigma-Aldrich, Germany) at 37°C for 20 min and then fixed in 4% paraformaldehyde overnight. The viable myocardium was red, while the infarcted myocardial tissues were pale.

### 2.5. H9C2 Cell Transfection and I/R Treatment

Rat myocardial cell H9C2 was grown in DMEM supplemented with 10% fetal bovine serum (FBS) and an environment with 5% carbon dioxide (CO_2_) at 37°C. The cells in logarithmic phase were transfected by shRNA-TGF*β*1 (OriGene, MA, USA; No. TG710434). The cells were subjected to I/R 72 h later. For I/R, the cells were grown in low glucose serum free DMEM to mimic ischemic condition.

### 2.6. Quantitative Real-Time PCR

Total RNA was extracted from mouse LV tissue and cells with TRIzol reagent (Invitrogen, USA). A total of 1 *μ*g of RNA was reversely transcribed into cDNA using the 5× PrimeScript RT kit (Takara, Japan). Quantitative real-time RT-PCR (qPCR) was performed with the SYBR green (Takara, Japan) in an ABI 7500 Fast (Applied Biosystems) with the following conditions: denaturation at 95°C for 10 s, annealing at 60°C for 5 s, and extension at 72°C for 8 s. TGF*β* expression was normalized to GAPDH expression. The primers used for quantitative PCR are presented in Table 1.

### 2.7. Western Blotting

The Protein Extraction Kit containing protease inhibitor and phosphatase inhibitor cocktails was employed to extract total proteins from heart tissue or cells according to the manufacturer's instructions. The protein concentration in each sample was determined by the BCA assay. Then, the protein lysates were mixed with 5× loading buffer and boiled at 100°C for 10 min. Subsequently, 20 *μ*g of protein was subjected to electrophoresis on the 10% SDS-polyacrylamide gels and then transferred onto PVDF membranes which were blocked with 5% (*w*/*v*) nonfat milk in TBST at room temperature for 1 h. The membranes were subsequently incubated with the following antibodies at 4°C overnight: anti-TGF*β*, anti-AKT1, anti-STAT3, anti-NF-*κ*B, anti-Smad2/3, anti-Smad4, anti-smad6, anti-smad7, anti-Bax, anti-BCL-2, and anti-caspase-3.

### 2.8. TUNEL Staining

The animals were killed at 24 h after surgery. The LV tissues were collected to prepare the frozen section. The section was fixed in 4% paraformaldehyde for 1 h at room temperature and incubated with Triton X-100 (0.1%) on ice for 2 min. Then, the section was rinsed with phosphate-buffered saline (PBS) solution thrice and incubated in FITC-marked TUNEL (50 *μ*l) at 37°C for 60 min. Finally, the section was observed under the fluorescence microscope.

### 2.9. Detection of Creatine Kinase (CK), Lactate Dehydrogenase (LDH), Aspartate Transaminase (AST), Malondialdehyde (MDA), Superoxide Dismutase (SOD), and Inflammation-Associated Cytokines

Blood samples were collected after reperfusion and then processed for the following measurements: serum levels of CK, AST, and LDH and inflammation-associated cytokines (such as tumor necrosis factor *α*, IL-1, and IL-6) were detected with corresponding ELISA kits based on the manufacturer's instructions.

### 2.10. Luciferase Report of TGF*β*

Total cDNA of H9C2 cells was used to amplify TGF*β*1 gene by PCR, which was then inserted into the pMIR-REPORT™ Luciferase (pMIR-R-L) control vector (Ambion). The Renilla luciferase vector was employed for normalization. The cells in 24-well plates were transfected with 0.2 *μ*g of pMIR-R-L vector and 0.04 *μ*g of control vector in the presence of Lipofectamine 2000. Moreover, pCMV6-ALK4 or inhibitor-ALK4 was used in each well. At 48 h after transfection, the dual-luciferase reporter assay system (Promega) was used to measure the pMIR-R-L and Renilla luciferase activities consecutively.

### 2.11. Statistical Analysis

Statistical analysis was performed with Statistical Product and Service Solutions version 23.0 (SPSS Inc., Chicago, IL, USA). The data are presented as mean ± standard deviation (SD). The comparisons were done with Student's *t*-test between groups and one-way analysis of variance among groups, followed by the Tukey-Kramer test. A value of two-sided *P* < 0.05 was considered statistically significant.

## 3. Results

### 3.1. Decreased ALK4 Expression Ameliorates Myocardial I/R Injury in Mice

To investigate the role of ALK4 in cardiac I/R injury, a cardiac I/R injury model was established in the ALK4^+/-^ transgenic mice. Ischemic reperfusion myocardial injury manifested in changes in myocardial enzyme profile, referred to as creatine kinase (CK), lactate dehydrogenase (LDH), and aspartate transaminase (AST), while manifesting changes in oxidative stress levels. The significant difference between MDA and SOD is determined.

**Figure 1:**
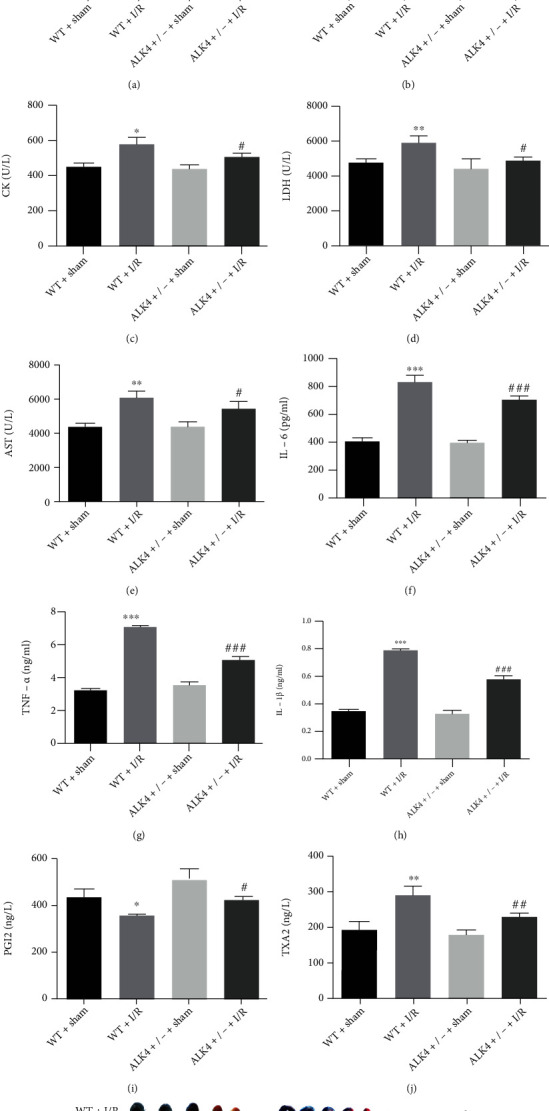
Serum levels of related factors in different groups: WT, ALK4-NC, ALK4-NC+I/R, and ALK4-KO+I/R groups. (a) The hydroxylamine method was used to detect SOD level at 24 h after I/R. (b) The thiobarbituric acid method was employed to detect MDA level at 24 h after I/R. (c–e) Serum levels of CK, LDH, and AST were assessed with corresponding ELISA kits at 24 h after I/R. (f–h) Inflammation related factors (IL-6, TNF-*α*, and IL-1*β*) were detected with corresponding ELISA kits at 24 h after I/R. (i and j) The serum levels of PG12 and TXA2 were detected with corresponding ELISA kits at 24 h after I/R. (k) Representative images of nonischemic zones (blue) and infracted tissue (white) within the AAR (absence of blue dye). Data are expressed as mean ± SD. ^∗^*P* < 0.05, ^∗∗^*P* < 0.01, and ^∗∗∗^*P* < 0.001, WT+I/R group vs. WT+sham group. ^#^*P* < 0.05, ^##^*P* < 0.01, and ^###^*P* < 0.001, ALK4^+/-^+I/R group vs. WT+I/R group.

As shown in Figure 1, compared to the WT+sham group, the serum SOD activity was decreased in the WT+I/R group (Figure 1(a)), whereas the serum levels of MDA (Figure 1(b)), CK (Figure 1(c)), LDH (Figure 1(d)), and AST (Figure 1(e)) and inflammation-related cytokines IL-6 (Figure 1(f)), TNF-*α* (Figure 1(g)), IL-1*β* (Figure 1(h)), and TAX2 (Figure 1(j)) significantly increased, and the serum PG12 level (Figure 1(i)) decreased at 24 h after I/R, and there were no marked differences in these indicators between ALK4^+/-^+sham group and WT+sham group.

In addition, mice experiencing myocardial I/R injury showed a bigger infarct size (Figure 1(k)), indicating that a successful I/R model was built. The serum levels of SOD decreased (Figure 1(a)), and those of MDA (Figure 1(b)), CK (Figure 1(c)), LDH (Figure 1(d)), AST (Figure 1(e)), IL-6 (Figure 1(f)), TNF-*α* (Figure 1(g)), IL-1*β* (Figure 1(h)), and TAX2 (Figure 1(j)) dramatically reduced in the ALK4^+/-^+I/R group, the infarct size reduced (Figure 1(k)), and the PG12 expression elevated (Figure 1(i)) as compared to the WT+I/R group. These results indicated that a decrease in ALK4 expression improves myocardial I/R injury in mice.

### 3.2. ALK4 Knockdown Inhibits Cardiac Cell Apoptosis and Downregulates TGF-*β* Signaling Pathway in Mice

**Figure 2:**
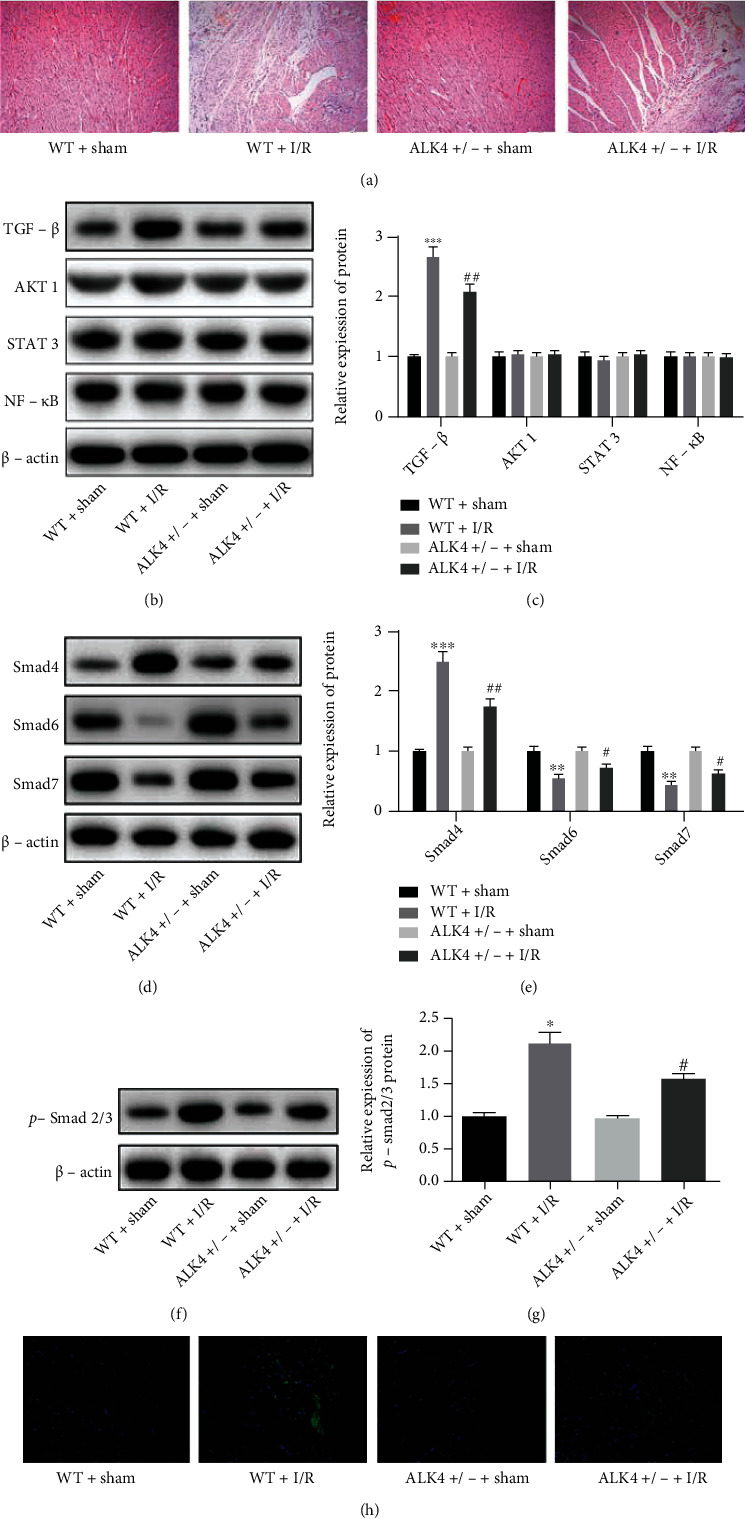
Detection of cell aging, cell apoptosis, and related molecules in different groups: WT+sham group, WT+I/R group, ALK4^+/-^+sham group, and ALK4^+/-^+I/R group. (a) HE staining of cell aging. (b and c) Detection of protein expressions of TGF*β*, AKT1, STAT3, and NF-*κ*B (Western blotting). (d–g) Detection of protein expressions of phosphorylated Smad2/3 (p-Smad2/3), Smad4, Smad6, and Smad7 (Western blotting). (h) Green: apoptotic cells (TUNEL staining); blue: nuclei (DAPI counterstaining, ×400). ^∗^*P* < 0.05 and ^∗∗^*P* < 0.01, WT+I/R group vs. WT+sham group. ^#^*P* < 0.05 and ^##^*P* < 0.01, ALK4^+/-^+I/R group vs. WT+I/R group.

To explore the mechanism underlying the cardioprotective effects of ALK4 knockdown on the myocardial I/R injury, the pathology of cardiac cells was evaluated. As shown in Figure 2(a), the tissues presented deeper colour when mice underwent I/R injury, and downregulation of ALK4 expression attenuated I/R injury. Western blotting was performed to detect the molecules related to cell proliferation in the TGF-*β* signaling pathway (such as TGF*β*1, AKT1, STAT3, and NF-*κ*B), but only TGF*β*1 expression was significantly different between groups (Figures 2(b) and 2(c)). The protein expressions of p-Smad2/3, Smad4, Smad6, and Smad7 related to the TGF-*β* signaling pathway were also detected. As shown in Figures 2(d)–2(g), the protein expressions of p-Smad2/3 and Smad4 in WT+I/R group increased significantly, and the expressions of Smad6 and Smad7 decreased markedly when compared with those in the WT + sham group. However, in the AKL4^+/-^ mice, these results were reversed. Additionally, TUNEL assay was performed to assess apoptotic cells. The results showed that the rate of apoptotic cells in the WT+I/R group increased significantly as compared to the WT+sham group; the rate of apoptotic cells reduced significantly in ALK4^+/-^ transgenic mice as compared to the WT+I/R group. These results indicate that ALK4 knockdown inhibits cardiomyocyte apoptosis via modulating TGF-*β* signaling pathway *in vivo*.

### 3.3. Decreased TGF-*β*1 Expression Improves Myocardial I/R Injury *In Vitro*

**Figure 3:**
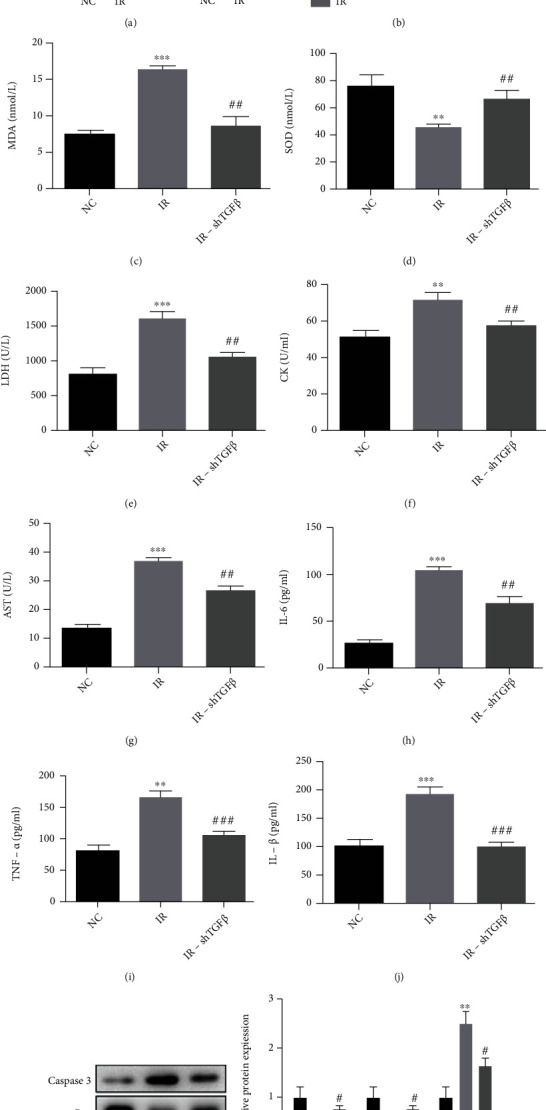
Expression of molecules related to TGF-*β* signaling pathway, antioxidants, inflammation-associated cytokines, and apoptosis-related molecules at 24 h after I/R and another 72 h after H9C2 cells were transfected with shRNA-TGF-*β*. (a and b) Expression of TGF*β*, p-Smad2/3, Smad4, Smad6, and Smad7 in the cells from the control group and I/R group at 24 h after I/R (Western blotting). (c) The thiobarbituric acid method was employed to detect MDA level. (d) The hydroxylamine method was used to detect SOD. (e–g) Serum levels of CK, LDH, and AST were detected with corresponding ELISA kits. (h–j) Inflammation-related factors (IL-1*β*, TNF-*α*, and IL-6) were detected with corresponding ELISA kits. (k and l) Expression of BCL-2, Bax, and caspase-3 (Western blotting). Data are presented as mean ± SD. ^∗^*P* < 0.05, ^∗∗^*P* < 0.01, and ^∗∗∗^*P* < 0.001, IR group vs. NC group. ^#^*P* < 0.05, ^##^*P* < 0.01, and ^###^*P* < 0.001, IR-sh TGF-*β* group vs. IR group.

TGF*β*'s direct treatment of H9C2 cells presents with exacerbation of myocardial damage and increases apoptosis of cardiomyocytes. Transforming growth factor *β* (TGF*β*) plays a vital role in regulating tissue homeostasis by controlling multiple cellular processes such as proliferation, differentiation, and matrix formation, by binding to different receptors of the activator receptor-like kinase (ALK) family, known TGF*β* induces receptor-regulated phosphorylation of the intracellular carboxyl terminal (C-terminus) of the Smad (R-Smad) protein, such as Smad1/2/3/5/8. Phosphorylated R-Smads form complexes with common medium SMAD (C-Smad), such as Smad4; in Smad4, these complexes can be transferred to the nucleus, where they bind to DNA, modulating gene transcription through the recruitment of transcription factors such as Cyclin D1, c-myc, and c-jun. There is growing evidence that TGF*β*1 is dysregulated in I/R damage to the heart, brain, kidneys, intestines, and other organs, suggesting that TGF*β*1 plays an important role in the occurrence and development of I/R damage. Then, the effect of TGF-*β*1 on the myocardial I/R injury was further investigated *in vitro*. Compared with the control group, the expressions of TGF*β*1, p-Smad2/3, and Smad4 increased significantly in the I/R group. However, the expressions of Smad6 and Smad7 reduced markedly (Figures 3(a) and 3(b)), which were consistent with findings from *in vivo* experiment. Then, shRNA targeting rat TGF-*β*1 was introduced to the cells to suppress the TGF-*β*1 expression. As shown in Figures 3(c)–3(j), the SOD expression increased, and the levels of MDA, CK, LDH, and AST and inflammatory-associated cytokines IL-6, TNF-*α*, and IL-1*β* all decreased when the cells were transfected with shRNA-TGF-*β*. The protein expressions of apoptosis-related proteins (such as Bax, Bcl-2, and caspase-3) were also detected by Western blotting. The results showed that, when compared with cells experiencing I/R, the Bcl-2 expression was upregulated, but the expression of Bax and caspase-3 decreased in the I/R-sh-TGF-*β* group (Figures 3(k) and 3(l)), indicating that downregulation of TGF-*β* expression inhibits apoptosis caused by I/R injury.

### 3.4. Downregulation of ALK4 Expression Ameliorates Myocardial I/R Injury through TGF-*β* Signaling Pathway *In Vitro*

Cell experiments and animal experiments observe different objects. Alk-ko IR was included in the study and compared to several other groups.

In the animal model, ischemia reperfusion model and sham surgical components WT-sham, WT+I/R, ALK+/-+sham, and ALK+/-+ I/R were established using wild-type mice and ALK knockout mice.

Two sets of controls were established in cellular hypoxia reoxygenation to simplify the experiments ALK4-NC+I/R and ALK4-KO+I/R.

**Figure 4:**
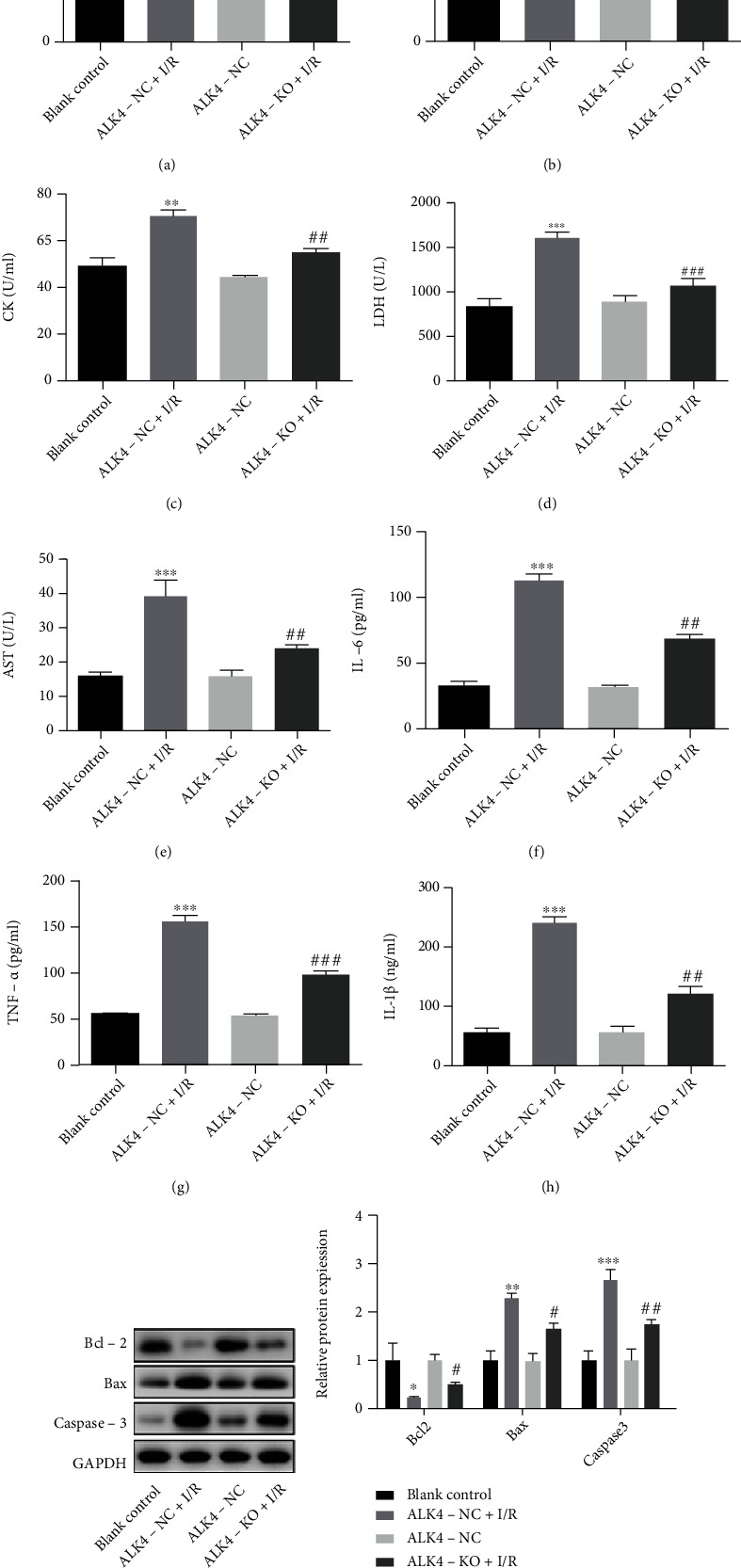
Expression of molecules related to TGF-*β* signaling pathway, antioxidants, inflammation-associated cytokines, and apoptosis-related molecules at 24 h after I/R injury in H9C2 cells. (a) The hydroxylamine method was used to detect SOD level. (b) The thiobarbituric acid method was used to detect MDA level. (c–e) Serum levels of CK, LDH, and AST were detected with corresponding ELISA kits. (f–h) Inflammation-related factors (IL-1*β*, TNF-*α*, and IL-6) were detected with corresponding ELISA kits. (i and j) Expression of BCL-2, Bax, and caspase-3 (Western blotting). Data are expressed as mean ± SD. ^∗^*P* < 0.05, ^∗∗^*P* < 0.01, and ^∗∗∗^*P* < 0.001, ALK4-NC+I/R group vs. ALK4-NC group. ^#^*P* < 0.05, ^##^*P* < 0.01, and ^###^*P* < 0.001, ALK4-KO+I/R group vs. ALK4-NC+I/R group.

To further explore the role of ALK4 in the cardiac I/R injury, the model of cardiac I/R injury was established in H9C2 cells with downregulation of ALK4 expression. As shown in Figure 4, the SOD decreased significantly (Figure 4(a)), and the levels of MDA (Figure 4(b)), CK (Figure 4(c)), LDH (Figure 4(d)), AST (Figure 4(e)), IL-6 (Figure 4(f)), TNF-*α* (Figure 4(g)), and IL-1*β* (Figure 4(h)) markedly increased in the ALK4-NC+IR group at 24 h after I/R injury as compared to the ALK4-NC group, and there were no significant differences between the blank control group and ALK4-NC group. In addition, H9C2 cells experiencing I/R injury showed a decreased Bcl-2 expression and elevated expression of caspase-3 and Bax (Figures 4(i) and 4(j)). Moreover, the expression of SOD increased (Figure 4(a)), and the serum levels of MDA (Figure 4(b)), CK (Figure 4(c)), LDH (Figure 4(d)), AST (Figure 4(e)), IL-6 (Figure 4(f)), TNF-*α* (Figure 4(g)), IL-1*β* (Figure 4(h)), and caspase-3 and Bax (Figures 4(i) and 4(j)) dramatically reduced at 24 h after I/R in the cells with downregulation of ALK4 expression. These results indicated that downregulation of ALK4 expression improves myocardial I/R injury *in vitro*.

**Figure 5:**
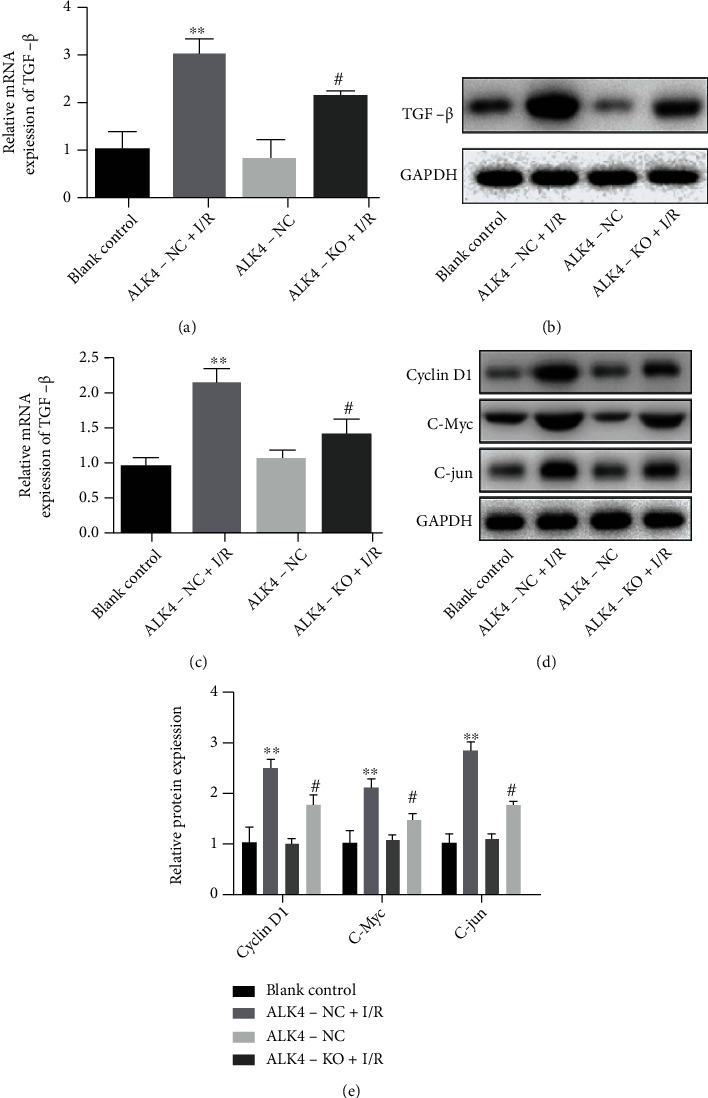
Effects of ALK4 on the expression of molecules related to TGF-*β* signaling pathway. (a–c) mRNA (PCR) and protein (Western blotting) expressions of TGF-*β* in different groups. (d and e) Expression of Cyclin D1, c-Myc, and c-Jun was influenced by I/R injury and ALK4 (Western blotting). Data are presented as mean ± SD. ^∗^*P* < 0.05, ^∗∗^*P* < 0.01, and ^∗∗∗^*P* < 0.001, ALK4-NC+I/R group vs. ALK4-NC group. ^#^*P* < 0.05, ^##^*P* < 0.01, and ^###^*P* < 0.001, ALK4-KO+I/R group vs. ALK4-NC+I/R group.

**Figure 6:**
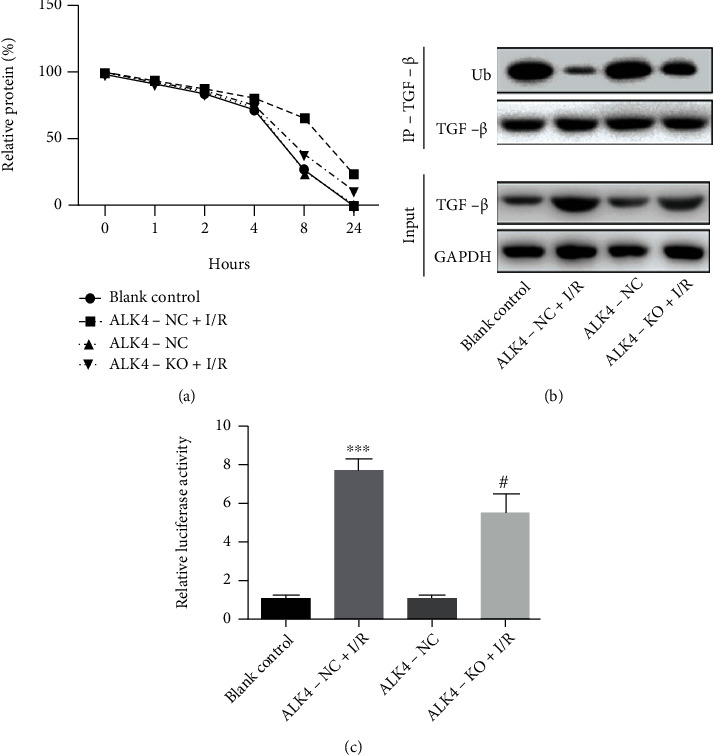
Effects of ALK4 on TGF-*β* protein stability and transcriptional activity in the H9C2 cells experiencing I/R injury. (a) CHX was used to inhibit protein synthesis. The cells were lysed and total proteins were extracted for Western blotting with anti-TGF-*β* antibody. (b) Coimmunoprecipitation assay of Ub protein expression. (c) Luciferase report assay was carried out to assess the transcriptional activity of TGF-*β* promoter. Data are presented as mean ± SD. ^∗^*P* < 0.05, ^∗∗^*P* < 0.01, and ^∗∗∗^*P* < 0.001, ALK4-NC+I/R group vs. ALK4-NC group. ^#^*P* < 0.05, ^##^*P* < 0.01, and ^###^*P* < 0.001, ALK4-KO+I/R group vs. ALK4-NC+I/R group.

The mechanism underlying the protective effects of ALK4 knockdown was further investigated in the cell model of I/R injury. Western blotting and quantitative PCR showed that the protein and mRNA expressions of TGF-*β*1 increased when the cells experienced I/R injury, while the TGF-*β*1 expression reduced when the cells with shRNA-ALK4 transfection experienced I/R injury (Figures 5(a)–5(c)). In addition, the expressions of CyclinD1, c-Myc, and c-jun increased in the I/R injury cell model, but they all decreased in cell ALK4 downregulation experiencing I/R injury (Figures 5(d) and 5(e)). Moreover, cycloheximide (CHX) was used to inhibit protein synthesis, and the protein was harvested for Western blotting, aiming to determine the stability of TGF-*β*1. As shown in Figure 6(a), the results indicated that TGF-*β*1 degradation was slowed down significantly in the cells experiencing I/R injury, which was alleviated after ALK4 downregulation in the cells experiencing I/R injury. Western blotting revealed that the Ub expression decreased in the ALK4-NC+I/R group, but the Ub expression increased after the expression of ALK4 was downregulated (Figure 6(b)), which was determined by luciferase report assay (Figure 6(c)), suggesting that downregulation of ALK4 expression most likely lowers TGF-*β*1 protein stability and transcriptional activity in the cells experiencing I/R injury.

**Figure 7:**
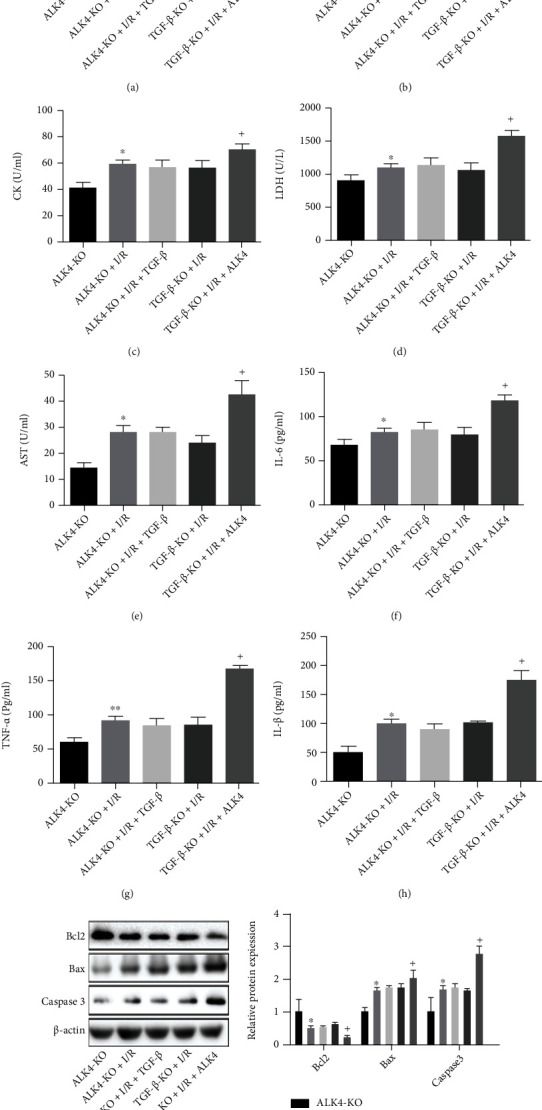
Knockout of ALK4 in the cell model of hypoxia/reoxygenation while exogenous TGF-*β* protein treatment. (a–e) Antioxidation indicators SOD, MDA, CK, LDH, and AST, and inflammatory response indicators TNF-*α*, IL-6, and IL-1. (i) WB detection Bcl2, Bax, and caspase-3.

**Figure 8:**
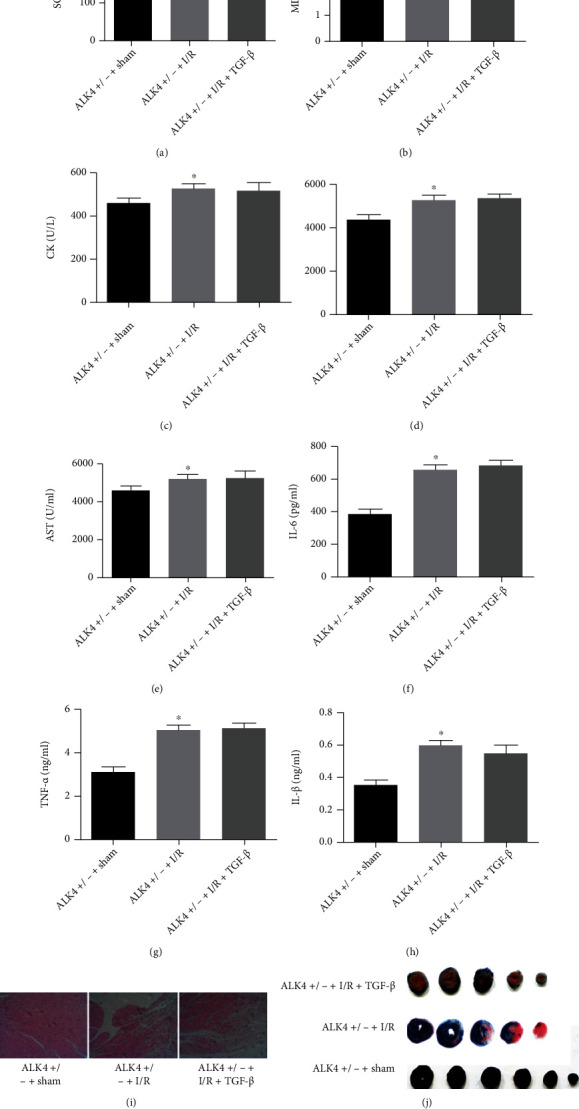
In the cell model of hypoxia/reoxygenation, TGF-*β* was knocked out, and exogenous ALK4 protein was added. HE staining of heart tissues. The aging condition: ALK4^+/−^ + I/R group = ALK4^+/−^ + I/R and TGFb group > ALK4^+/−^ + sham group. Evans blue and TTC staining found no significant change in myocardial infarction area after addition of TGF-*β*, followed by detection of SOD, MDA, CK, LDH, AST, IL-6, TNF-*α*, and IL-1*β*. There was no significant difference between ALK4^+/-^+I/R and TGF-*β* groups, and it was significantly higher than the ALK4^+/-^+sham groups. (a–h) Antioxidation-related indicators and inflammation-related indicators TNF-*α*, IL-6, and IL-1. (i) HE staining. (j) Evans Blue and TTC double staining.

Figures 7 and 8 are further illustrative of the bidirectional relationship between exogenous TGF*β* and ALK4. In the cell model of hypoxia/reoxygenation, ALK4 was knocked out, and exogenous TGF protein was added. Our results showed the I/R injury was alleviated after knocking out ALK4, and after the administration of exogenous TGF-*β*, there was no marked difference between I/R injury group and ALK4 knockout group. This suggests that ALK4 knockout improved cell injury, which did not disappear after addition of TGF-*β* protein (Figure 7).

In the cell model of hypoxia/reoxygenation, TGF-*β* was knocked out, and exogenous ALK4 protein was added. The results showed that the ischemic reperfusion injury was alleviated after TGF-*β* knockout and after external ALK4. There is a significant difference between the injection damage group and TGF-*β* knockout group. In addition, the improvement of cell injury after TGF-*β* knockout decreased after addition of ALK4 protein, and thus, these findings indicate that TGF-*β* knockout reduces the ALK4 expression to relieve cell injury (Figure 8).

## 4. Discussion

The reperfusion after myocardium ischemia is a pivotal therapeutic strategy used to alleviate ischemic symptoms and avoid more extensive injury [22]. However, it was shown that 50% of the final myocardial infarction (MI) area may occur in I/R damage [23]. In this study, our results showed that ALK4 knockout alleviated the myocardial I/R injury through inactivating TGF-*β* and Smad2/3 signaling both *in vivo* and *in vitro*. In addition, accumulating evidence suggests that the increases in the TGF superfamily ligands are associated with the progression of heart failure. Moreover, the levels of ALK4 and ALK5 ligand TGF were detected both in heart failure patients and animals with heart failure, and the results showed the levels of these proteins were positively related to the disease severity. Heart failure patients often develop a progressive deterioration of cardiac function, which may be ascribed to the pathological remodeling due to the increased hemodynamic stress. Cardiac remodeling is generally regarded as a sign of irreversible heart disease and closely associated with a poor prognosis. Currently, treatments are employed mainly to slow the functional decline of the heart but may not prevent or reverse the progression of the disease. These suggest that ALK4/TGF*β*/Smad2/3 pathway is a maladaptive factor in case of myocardial I/R injury and therefore inhibition of ALK4 may become a novel therapeutic and prophylactic target of myocardial I/R injury.

ALKs transduce TGF-*β* signals to regulate a lot of cellular processes (such as proliferation, differentiation, apoptosis, adhesion, and migration), and therefore, they play crucial roles in a variety of biological processes [24]. In addition, some studies have found the function of ALKs in the cardiac expression by using gene manipulation approaches [25, 26]. Shahid et al. [26] reported that ALK2 was involved in the angiotensin II-induced cardiac remodeling; the maladaptive changes were reversed and the left ventricular systolic function was restored after cardiac-specific deletion of ALK2. Huang et al. [25] reported that the ALK7 gain-of-function inhibited cardiac hypertrophy and fibrosis, exerting cardioprotective effects in the mice. Li et al. [27] suggested that partial inhibition of ALK4 attenuates cardiac fibrosis induced by pressure overload and improves cardiac function, which implies that inhibition of ALK4 plays a cardioprotective role. In a previous study from our center, our results showed that haplodeficiency of ALK4 alleviated cardiac fibrosis secondary to MI and retained cardiac function [20]. Our study investigated the role of ALK4 knockdown on the myocardial I/R injury, and the results showed that ALK4 knockout ameliorated the I/R injury in mice. In this study, we show that TGF-*β*1 is elevated in the I/R model and downregulation of TGF-*β*1 expression protected the myocardium from inflammatory and oxidative injuries after I/R. TGF-*β* signaling has been described to have both good and bad roles in heart remodeling [28]. For example, DeBerge et al. [29] recently reported that MerTK cleavage on the resident cardiac macrophages compromised the repair after myocardial I/R injury through inhibiting the secretion of proreparative factors, including TGF-*β*, which indicates the cardioprotective role of TGF-*β*1. However, studies have also shown that TGF-*β*1 promotes myocardial fibrosis, cardiomyocyte apoptosis [30], and cardiac hypertrophy [31]. In addition, an important relationship between reduced ejection fraction in patients after AMI and increased TGF*β* expression has also been reported [32], demonstrating the detrimental effects of TGF-*β* in the heart. In this study, our results showed the detrimental effects of TGF-*β*1 on the myocardial I/R injury, and the overexpression of TGF-*β* increased the levels of IL-6, SOD, TNF-*α*, IL-1*β*, MDA, CK, LDH, AST, and TAX2, promoted cell apoptosis, and aggravated I/R injury.

In the present study, the expression of Smad2/3 and Smad4 was significantly downregulated after ALK4 knockdown in the H9C2 cells, followed by inactivation of Cyclin D1, c-jun, and c-Myc, through which ALK4 may exert effects on the progression of I/R injury. The individual ligand of TGF-*β* superfamily may transmit signals after binding to its cognate type II transmembrane serine/threonine kinase receptor. Then, the activated type II receptors pass through seven type I receptors (ALK1-7). Both TGF-*β* and activin phosphorylate Smad2/3 [33]. The phosphorylated Smad2/3 subsequently binds to Smad4, forming Smad2/3/4 complex which translocates from the cytoplasm into the nucleus. Through activating Smad2/3 signaling pathway, ALK4/5 ligands can regulate a lot of processes, including tissue repair, inflammation, cell proliferation, and differentiation [34].

The potential mechanism of ALK4 in I/R damage cell models and effects of ALK4 on protein expression levels were associated with TGF signal transduction pathways. Western blotting and QPCR results show that the protein and mRNA expression levels of TGF*β*1 increase when the cells are treated with H/R damage, while the expression levels of TGF*β*1 decrease when the cells are treated with shRNA-ALK4. In addition, H/R damage cell models showed increased expression levels of CyclinD1, c-Myc, and c-jun, but their levels decreased after downregulating ALK4 expression in I/R damage cell models. In addition, we use cycloheximide (CHX) to stop protein synthesis over a specified period, harvest proteins, and perform Western blot analysis to determine the stability of TGF*β*1. As shown, the results show that TGF*β*-*γ* degradation slows down significantly when treating cells with H/R damage, and this trend is mitigated by downregulating ALK4. The Western blotting results showed a decrease in the expression of Ub in ALK4-NC+IR cells, while an increase in the expression of Ub at the time of the current regulation of ALK4, as well as the transcriptional activity of TGF*β*, which was measured by luciferase reporting assay, indicating that decreased ALK4 levels are most likely to reduce the stability and transcriptional activity of TGF*β* proteins in I/R-damaged cells. These results suggest that a decrease in ALK4 levels plays a role in improving myocardial I/R damage through in vitro TGF*β* signaling.

Our results showed that downregulation of ALK4 expression decreased TGF-*β*1 expression; however, if ALK4 interacts with ALK5 through TGF-*β* as TGF-*β* is a ligand of ALK5 remains unknown, which needs to be further studied. Furthermore, there is evidence showing that ALK4 mediates signals initiated by some members of TGF-*β* superfamily (such as activin and myostatin) [24]. However, the TGF-*β* signal transduction pathway must have the characteristics of continuous, strict regulation of signal strength, and its regulatory mechanism requires multiple functions such as (positive/negative) feedback regulation. There are different regulatory mechanisms in different cells or under physiological conditions, and the final information output of the signaling pathway is the result of a combination of various regulatory mechanisms. TGF-*β* signaling pathways are regulated by a variety of mechanisms at ligand, receptor, and Smad levels. Our results showed that the ALK4 downregulation reduced the expression of TGF-*β*1. However, if ALK4 could interact with ALK5 through TGF-*β* should be further studied. In the present study, only the effect of ALK4 on the TGF-*β* expression was explored, but which upstream ligand of the TGF-*β* superfamily that functionally binds to the type II receptor in the heart and then activates ALK4 signaling cascade is not investigated. Further studies are required to address this important issue.

## 5. Conclusions

This study explores the underlying mechanism underlying the effects of ALK4 on the myocardial I/R injury both *in vivo* and *in vitro*. Our results indicate that ALK4 knockdown is able to improve myocardial repair after myocardial I/R injury. Thus, ALK4 might be used as a novel target for the therapy and prevention of myocardial I/R injury.

## Figures and Tables

**Table 1:** The primers used for quantitative PCR.

Gene name	Primer sequence
TGF*β* forward	5′-GCTGAACCAAGGAGACGGAA-3′
Reverse	5′-GGATCCACTTCCAACCCAGG-3′
GAPDH forward	5′-CATCACCATCTTCCAGGAGCG-3′
Reverse	5′-TGACCTTGCCCACAGCCTTG-3′

## Data Availability

The data that support the findings of this study are available on request from the corresponding author.
